# Effects of Aging Torque Controllers on Screw Tightening Force and Bacterial Micro-Leakage on the Implant-Abutment Complex

**DOI:** 10.3390/ma15020620

**Published:** 2022-01-14

**Authors:** Yousef Jiries, Tamar Brosh, Shlomo Matalon, Vladimir Perlis, Zeev Ormianer

**Affiliations:** Department of Oral Rehabilitation, The Maurice and Gabriela Goldschleger School of Dental Medicine, Tel Aviv University, Tel Aviv 6997801, Israel; yousefjiries1@gmail.com (Y.J.); tbrosh@tauex.tau.ac.il (T.B.); matalons@tauex.tau.ac.il (S.M.); doctorperlis@gmail.com (V.P.)

**Keywords:** torque, torque controller, sterilization, bacteria penetration

## Abstract

Aim: We assess the accuracy of torque controllers after several aging processes and the bacterial leakage on implant-abutment complexes (IAC). Methods: A total of 12 spring-type and 12 friction-type torque controllers and 48 IAC (24 conical and 24 hexagonal connections) were evaluated. Chemical, mechanical, temperature, and pressure-aging methods were applied individually to replicate clinical use. Torque controller accuracy was analyzed before and after aging using a calibrated gauge. To assess bacterial leakage, the IAC were suspended in a bacterial medium for 24 h. Direct Contact Test (DCT) and Polymerase Chain Reaction Test (RT-PCR) analyzed the infiltration of *F. nucleatum* and *P. gingivalis* into the IAC micro-gap. Results: A significant decrease in torque after 10 days of aging was found. The spring-type torque controller was affected the most, regardless of the aging method (*P* < 0.05). PCR results indicated that all groups exhibited significantly more bacterial leakage, regardless of the method used (*P* < 0.05). The conical IAC demonstrated more bacterial leakage of *P. gingivalis* compared with the hexagonal IAC (*P* = 0.07). DCT found bacterial growth in the IAC only before aging and was not identified after aging. Conclusion: Aging affects torque accuracy. A reduction in force was noticed after 10 days. The conical IAC exhibits more bacterial leakage, although this was not statistically significant.

## 1. Introduction

Dental implants are a common solution for edentulous ridges. Unintentional loosening of the implant/abutment complex is a frequent problem related to dental implants [1,2]. To avoid complications, it is important to use controlled torques for fixating abutments on implants [3]. Applying torque to the abutment screw complex generates a tension force known as preload on the screw [4]. Under-torquing the screw joint causes dynamic fatigue and greater micro-motion at the implant-abutment junction, resulting in failure and loss of function [5].

The maximum torque that can be applied on the implant-abutment screw is up to 120% of the torque recommended by the manufacturer [6]. Over-torquing can lead to screw deformation, thread stripping, screw loosening, and fracture [4,6,7].

Inaccurate torque can be applied to the fastening screws because of device condition, frequency of use, debris in the operating mechanism, and possible corrosion [8,9].

Mechanical torque controllers (MTC) have several designs, among them are coil design, toggle design, and spring design.

The accuracy of the devices is within 10% of the target torque [10]. Therefore, torque-limiting devices, particularly friction-style devices, should be checked and calibrated during clinical use [10,11].

One of the variables that can affect MTC accuracy is sterilization. A study analyzed the effect of steam sterilization on spring-style MTC and found that the accuracy of the devices after sterilization was within 10% of the target torque value [12]. Another study showed that sterilization with or without dismantling did not affect the accuracy of spring-style MTCs [13].

On the contrary, one study found that a statistically significant difference in the accuracy of friction-style MTC emerged when sterilization procedures and the number of uses were considered [14]. Low preload and micro-movements of the implant-abutment surface may result in bacterial penetration into the implant [5].

Implant-abutment micro-movements can induce bacterial infiltration, which can lead to bone loss around this area [5,15,16,17]. The amount of bacterial infiltration between the implants and the abutments depends on factors such as the fit accuracy between the implant and the abutment, and the tightening torque applied by MTC [17,18]. Several studies suggest that the interface micro-gap between the implant and the abutment can allow fluids to pass between them regardless of the implant system [17,18,19].

Bacterial infiltration was evaluated from inside the screw role to the outside and vice versa [17,20]. None of the studies quantified the number of bacteria that infiltrated inwardly to the implant-abutment interface.

**Figure 1:**
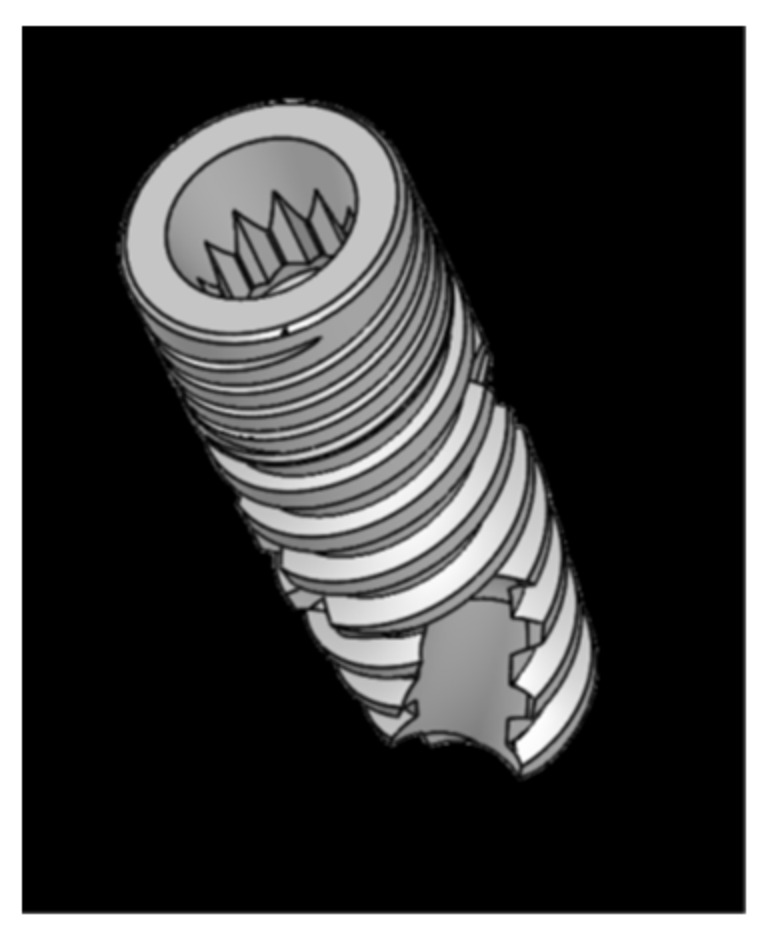
Conical connection.

**Figure 2:**
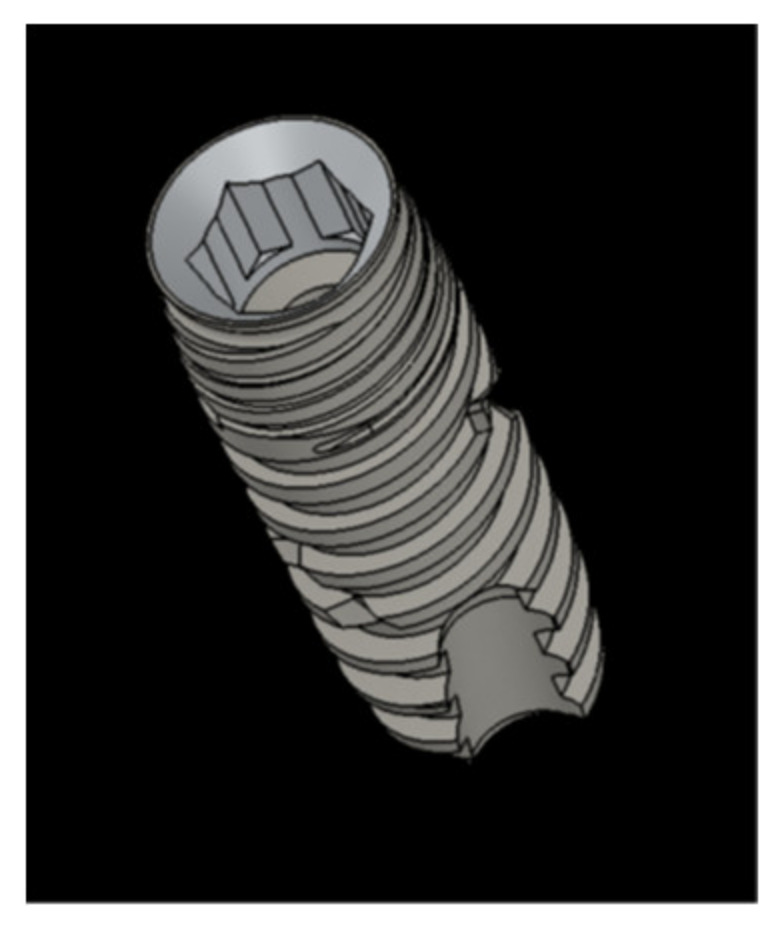
Hexagonal connection.

The design of the implant-abutment interface can have an impact on the amount of microbial penetration into the internal part of dental implants. The commonly used connections are the conical and internal hexagonal types (Figure 1 and Figure 2).

The conical connection type exhibits 21.9° angulation and 2.8 mm diameter. The hexagonal connection exhibits an internal depth of 2.0 mm.

In one study on the implant-abutment interface of external hexagon, implants on the tightening torque had no statistical influence on bacterial micro-leakage. However, only the samples that were tightened at 32 Ncm showed no bacterial leakage, compared with implants with less tightening force [21]. In a study that compared two implant-abutment connections, the conical connection type exhibited significantly better torsion strength and a smaller micro-gap than the mandatory internal hexagonal connection [17,22].

Some studies did not find statistical differences in the bacterial penetration into the implant/abutment complex between the internal hex and taper connections [23,24]. Conversely, other researchers showed that bacterial species from human saliva penetrated the internal hex connection implants significantly more than the morse cone connection [25,26,27].

The significance of aged torque controllers on bacterial leakage to different IAC types is not clearly addressed in the literature. Sterilization methods can induce more bacterial penetration and influence marginal bone loss around dental implants.

The purpose of this study is to evaluate the effect of chemical sterilization and autoclave sterilization on the accuracy of torque controllers and on the inward bacterial leakage onto abutment-implant complexes. The hypotheses are that the tightening torque activated by MTC correlates inversely with the number of uses and the number of sterilizations performed. It may affect bacterial leakage onto the IAC.

## 2. Materials and Methods

A total of 12 spring-type and 12 friction-type (Cortex Dental Implants Ltd. Shlomi, Israel) torque controllers were tested. In addition, 48 implants (Cortex Dynamix 8 mm implants and 5 mm metal straight abutments) were inserted in a special PEEK material mold (6 implants in each mold) to prevent any movement while tightening. The study was conducted by simulating the average monthly clinical use of the torque controllers, including tightening and sterilization. Average monthly clinical use was defined as tightening 9 abutments per day, over 30 days, with or without sterilization.

Five study groups were evaluated:

- Control: Tightening measurements of torque controllers before tightening the abutments and the aging processes;
- Mechanical aging: Tightening the system to simulate clinical use without sterilization;
- Chemical aging: Tightening the system to simulate clinical use plus chemical sterilization after tightening, by immersing the devices in 2% phenol and an aldehyde-free, non-fixing disinfectant (Deconex 53 plus, Borer Chemie, Zuchwil, Switzerland) for 20 min;
- Temperature and pressure aging: Tightening the system to simulate clinical use plus autoclaving for 15 min at 135 °C after tightening;
- Combined aging: Tightening the system to simulate clinical use, plus applying chemical sterilization after tightening. Moreover, it was autoclaved at every tightening over a period of 30 days to simulate clinical use.

Torque tightening was measured three times for each controller with a torque gauge (BTG60CN-S 500245G, Tohnichi Mfg. Co., Ltd., Tokyo, Japan) before the aging process. The results served as the control values for comparison to torque tightening during and after aging.

Two bacterial strains were used in our study: *F nucleatum* ATCC 1594 and *P. gingivalis* ATCC 33279.

### 2.1. Experimental Design

A total of 48 implant-abutment complexes were divided into 8 molds of 6 implants each (6 conical and 6 hexagonal connections). Each mold was assigned a number, and the number of each implant was the same as that of the specific torque controller device used.

Molds 1–4 included internal hexagonal implants, and molds 5–8 included internal conical implants.

In molds 1 and 5, the implants were numbered from 1 to 6. Six spring-style torque controller devices were used and numbered, with each device ascribed to two implants. These molds were placed in a suspension containing P. gingivalis.

Implants 1, 2, and 3 were assigned to Group C—chemical aging. Moreover, the devices ascribed to implants 4, 5, and 6 were assigned to Group B—mechanical aging.

In molds 2 and 6, the implants were numbered from 7 to 12. Six spring-style torque controller devices were used and numbered, with each device ascribed to two implants. These molds were placed in an F. nucleatum suspension. Implants 7, 8, and 9 were assigned to Group E—combined aging. Additionally, the devices ascribed to implants 10, 11, and 12 were assigned to Group D—temperature and pressure aging.

The implants in molds 3 and 7 were numbered 1–6. Six friction-style torque controller devices were used and numbered; each was assigned to two implants. These molds were placed in an F. nucleatum suspension. Implants 1, 2, and 3 were assigned to Group C—chemical aging. Additionally, the devices ascribed to implants number 4, 5, and 6 were assigned to Group B—mechanical aging.

In molds 4 and 8, the implants were numbered 7–12. Six friction-type torque controller devices were numbered, with each device ascribed to two implants. These molds were placed in a *P. gingivalis* suspension. Implants 7–9 were assigned to Group E—combined aging. Additionally, implants 10–12 were assigned to Group D—temperature and pressure aging (Table 1).

Each IAC was tightened using its specifically marked torque controller device and then aged according to its assigned aging group.

Subsequently, each group of six complexes was suspended in a 50 mL test tube containing 37 mL of growth medium suitable for each bacterial type:

*F. nucleatum*: Sherdler broth (Becton, Dickinson and Company, Franklin Lakes, NJ, USA).
*P. gingivalis*—Wilkins Chalgren Anaerobe broth (Oxoid Ltd., Hampshire, England).

One milliliter of bacterial suspension (optical density (OD) 0.6 at 650 nm) was added to each well, and the tubes were incubated in an anaerobic environment at 37 °C for 48 h.

Subsequently, the abutment was removed from each complex, and the inner thread of the implant was filled with 12 µL of a sterile growth medium, followed by a 60-second vortex to ensure homogeneous cell suspension. The suspension was then transferred to a specific well containing 220 µL of sterile growth medium in a flat-bottom 96-well (Nuclon, Nunc, Copenhagen, Denmark). This procedure was conducted twice, each time for one specific abutment connection.

The plate was divided as follows: six wells in a row (A1, A2, A3, A4, A5, and A6) were assigned to molds 1 and 5. Every well was therefore filled with 220 μL of sterile growth medium (Wilkins broth). Later, the 12 µL from the inner thread of the implants was added. Six wells in a row (C1, C2, C3, C4, C5, and C6) were assigned to molds 4 and 8. Accordingly, every well was filled with 220 μL of sterile growth medium (Wilkins broth). Later, the 12 µL from the inner thread of the implants was added. Three wells in a row (A10, A11, and A12) were filled with 220 µL sterile growth medium (Wilkins broth). After that, 12 µL of *P. gingivalis* was added, serving as a positive control. Three wells (A9, B9, and C9) were filled with 220 µL of sterile growth medium (Wilkins broth), serving as a negative control. Six wells in a row (E1, E2, E3, E4, E5, and E6) were assigned to molds 2 and 6. Therefore, every well was filled with 220 µL of sterile growth medium (Sherdler broth). Later, the 12 µL from the inner thread of the implant was added. Six wells in a row (G1, G2, G3, G4, G5, and G6) were assigned to molds 3 and 7. Consequently, every well was filled with 220 µL of sterile growth medium (Sherdler broth). Later, the 12 µL from the inner thread of the implant was added. Three wells in a row (E10, E11, and E12) were filled with 220 µL of sterile growth medium (Sherdler broth). After that, 12 µL of F. nucleatum was added, serving as a positive control. Three wells (E9, F9, and G9) were filled with 220 µL of the sterile growth medium (Sherdler broth), serving as a negative control.

The kinetics of the outgrowth from each well were recorded by measuring the OD at 650 nm every 30 min for a period of 24 h, using a temperature-controlled spectrophotometer (Versamax, Molecular Devices Corporation, Menlo Park, CA, USA) set at 37 °C. A 10-second vortex prior to each read ensured a homogeneous cell suspension.

The direct contact test (DCT) optical density values were analyzed, enabling the calculation of two parameters: (i) the slope of the linear portion of the growth curve, which expressed changes in bacterial growth rate and (ii) the distance of the ascending portion of the growth rate from the Y-axis, which correlated with the number of viable microorganisms at time zero. To ensure the measurements would be taken in an anaerobic environment, the spectrophotometer was moved into an anaerobic hood. Furthermore, after cell lysis was carried out for the bacteria, RT-PCR was performed to quantify the number of bacteria.

### 2.2. Statistical Analysis

One-way ANOVA, two-way ANOVA, four-way ANOVA, paired sample *t*-test, and Tuckey multiple comparisons were applied to the data to determine the associations between the bacterial infiltration and the aging processes. To test the difference in the median between the groups, the Mann-Whitney U test was used, according to a sample size calculation that considers test significance = 0.0166, power = 0.8, and effect size = 1.67.

## 3. Results

The torque measurements for the control group during the aging periods were higher than the pre-calibrated value of 30 Ncm (Table 2).

**Figure 3:**
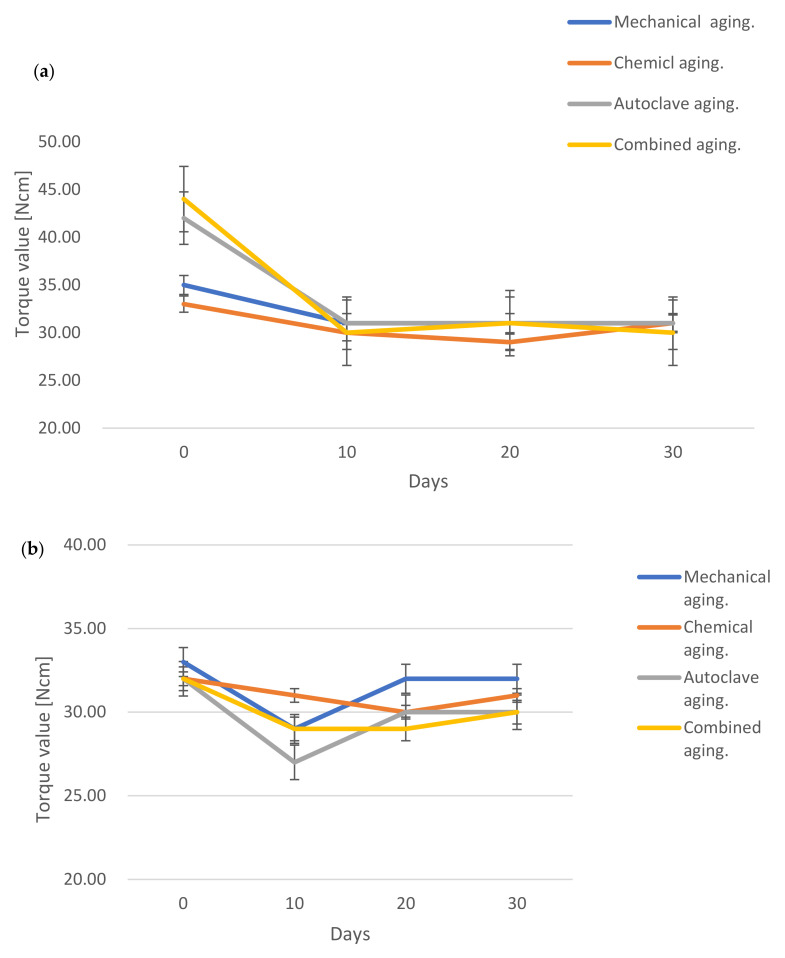
Effect of different aging processes after 10, 20, and 30 days on the accuracy of torque applied by spring-style torque controller devices (**a**). Effect of different aging processes after 10, 20, and 30 days on the accuracy of friction-style torque controllers whereas the X-axis = days and the Y-axis = mean torque value (**b**).

A significant decrease in the torque occurred after 10 days of aging for all methods (*P* = 0.012, Figure 3a,b).

Figure 3a shows a greater (though not significant) decrease in the combined category, followed by the autoclave, mechanical, and chemical aging categories. Figure 3b demonstrates a greater (though not significant) decrease in the autoclave category compared with the other categories, followed by the mechanical, combined, and chemical aging categories.

Autoclaving had the greatest effect on reducing the tightening force. Aging reduced the force on the spring-style compared with the friction-style torque controller, regardless of the method (*P* < 0.05).

The PCR results indicated that all experimental groups exhibited significantly more bacterial penetration than the control group, regardless of the aging method (Table 3).

A four-way ANOVA demonstrated that the type of bacteria affected penetration. *P. gingivalis* penetrated both IACs more regardless of the type of torque controller (Table 4).

*P. gingivalis* penetrated the conical IAC more in comparison to the hex connections that were tightened the same way (*P* = 0.07, Table 5).

**Figure 4:**
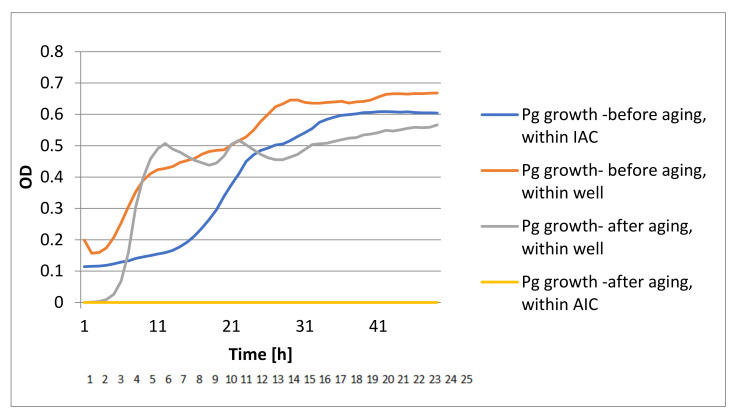
Conical DCT results of Pg bacterial growth.

**Figure 5:**
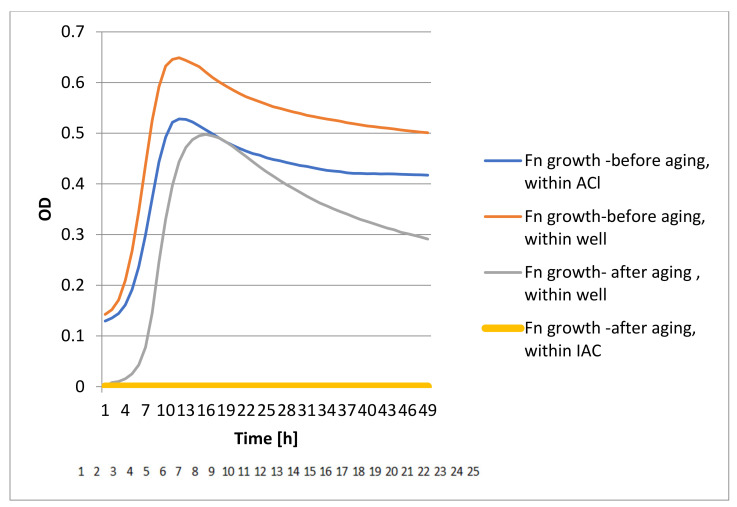
Conical DTC results of Fn bacterial growth.

**Figure 6:**
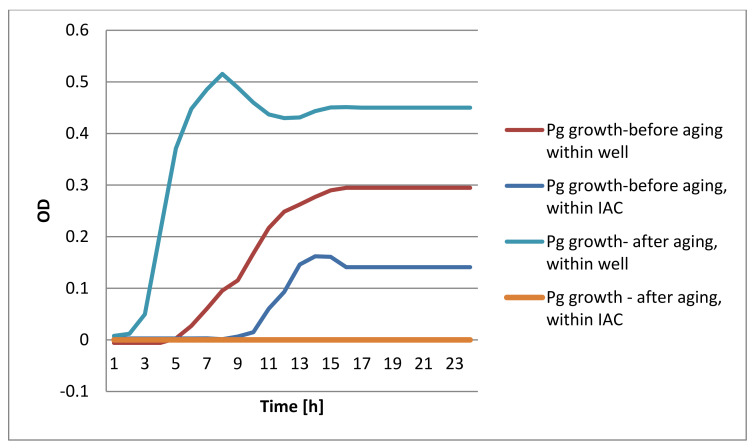
Hexagonal DCT results of Pg bacterial growth.

**Figure 7:**
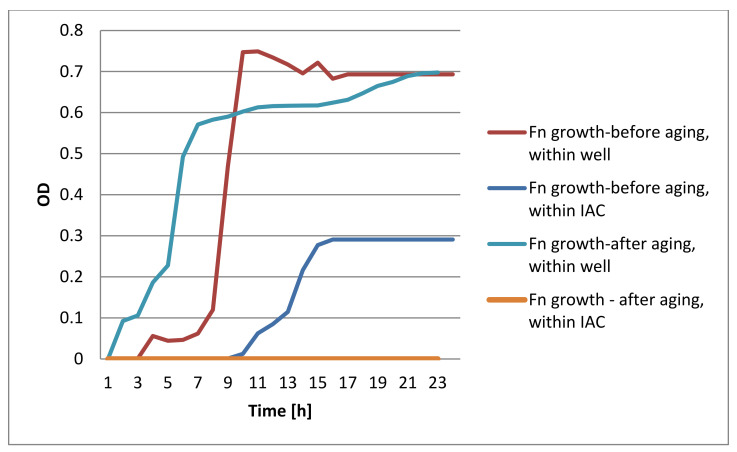
Hexagonal DCT results of Fn bacterial growth.

The DCT test showed bacterial growth in the pre-aged devices while no bacterial growth was identified in the aged devices (Figure 4, Figure 5, Figure 6 and Figure 7).

## 4. Discussion

A significant decrease in torque values within the first 10 days of the aging processes was observed, regardless of the method. However, there were no significant decreases in the measured torque values in the subsequent 20 days. Other studies showed that the aging processes did not significantly affect device accuracy, but no other information was given about the aging procedure [28,29,30]. Studies that examined friction-style devices showed that mechanical aging affects accuracy. While this conclusion supports our findings, it does not refer to other aging methods [29,30,31].

The study results showed that aging methods affected the spring-style devices more than the friction-style devices. The results of two other studies differed, showing that sterilization methods affected friction-style devices more than spring-style devices. The difference in the results could be explained by different aging methods used in each study [32,33].

Dellinges et al. showed that the autoclave and chemical sterilization processes did not affect the torque values of the torque controller devices after 100 cycles [9].

The current study shows that sterilization affects the accuracy of the devices. The mechanical aging conducted with all the aging types may explain the difference.

Bacteria penetration, especially of the *P. gingivalis* type, was found in this study. In previous studies, experiments were conducted using several types of bacteria. In part of the studies, E. coli was used because it is a widely used microorganism for in vitro studies, is easy to handle, and has a short generation time [17,21,34]. Others conducted their experiment using *Fusobacterium nucleatum* [18], *Porphyromonas gingivalis* [35], and *Staphylococcus aureus* [20]. In contrast, Gross et al. did not use any bacteria but instead used a dye to detect outward leakage from the implant-abutment complex [19].

*Fusobacterium nucleatum* is one of the most studied bacteria implicated in periodontal disease. It belongs to the *Bacteroidaceae* family and is a dominant micro-organism within the periodontium. It is a Gram-negative anaerobic species of the phylum. *Fusobacterium nucleatum* is numerically dominant in dental plaque biofilms. It is important in biofilm ecology and human infectious diseases [18].

*Porphyromonas gingivalis* is a Gram-negative anaerobic bacterium [24]. Infection by *P. gingivalis* can modulate host immuno-inflammatory responses and ultimately destroy the balance between the normal cell cycle and apoptosis, thereby leading to periodontal tissue destruction [13,18,36].

Information is limited regarding the correlation between the different aging methods and bacterial infiltration, in particular chemical aging. Furthermore, most studies examined the outward infiltration of the bacteria, from the implant to the outside environment. Those that did examine the inward infiltration did not quantify the number of infiltrated bacteria. However, bacterial leakage is considered to be less in conical IAC [37].

The IAC type has a significant impact on bacterial leakage. Several studies found bacterial leakage from conical connections [13,36,37,38]. Koutouzis et al. suggested that differences in implant design may affect the potential risk for colonization of oral microorganisms into the implant-abutment interface micro-gap. They found that the morse taper connection, which is a conical connection, exhibited significantly lower numbers of colony-forming units (CFU) compared with the four-groove conical internal connection [34]. Da Silva-Neto et al. suggested that morse taper connection is more effective in preventing microleakage [20].

Bacterial leakage was measured in a study with four implants that were immerged in a bacterial culture for 24 h. The number of bacteria was assessed inside the implant-abutment interface with real-time PCR. Bacteria were detected inside all studied implants, with a median percentage of 6% for *P. gingivalis* [26]. These results supported the leakage detected in the current study.

On the contrary, the conical implant complex bacterial infiltration was assessed in an in vitro study by tightening the abutment to the implant according to the manufacturer’s instructions and by immersing 10 IAC in bacterial broth for 24 h. No bacteria were detected using scanning electron microscopy. However, the aging processes were different from those used in the current study [13].

The DCT test showed no bacterial growth, which may be due to the repetitive tightening. Metal nanoparticles from the screw may sink into the apical portion of the implant, stimulating an antibacterial effect on the penetrating bacteria and thus preventing growth [38].

The limitations of this study include the short aging period. If the devices had been aged for a longer period, the effect might have been larger. Moreover, it could not be determined whether the bacteria penetrated through the implant-abutment complex gap or the abutment’s screw thread.

## 5. Conclusions

The aging process decreases the accuracy of torque controller devices regardless of the sterilization methods. A reduction in force was noticed after 10 days. The conical IAC exhibits more bacterial leakage, although this was not statistically significant. *P. gingivalis* penetrated IAC more than *F. nucleatum*.

## Figures and Tables

**Table 1:** The distribution of the experimental groups in each mold.

Mold Number	Type of Implant Connection	Type of Torque Controller	Implant Number	Type of Aging	Bacteria	Group
1	Hex	Spring	1–3	Chemical	*P. gingivalis*	C
5	Conical	Spring	4–6	Mechanical	*P. gingivalis*	B
2	Hex	Spring	7–9	Combined	*F. nucleatum*	E
6	Conical	Spring	10–12	Temperature Pressure	*F. nucleatum*	D
3	Hex	Friction	1–3	Chemical	*F. nucleatum*	C
7	Conical	Friction	4–6	Mechanical	*F. nucleatum*	B
4	Hex	Friction	7–9	Combined	*P. gingivalis*	E
8	Conical	Friction	10–12	Temperature Pressure	*P. gingivalis*	D

**Table 2:** The mean of friction-style and spring-style torque controller for tightening torque measurements (Ncm). The mean torque measurement results of each torque controller before aging and after 10, 20, and 30 days of aging (N = 24, *P* = 0.012).

Device	Aging Method	Control ± SD	Day 10 ± SD	Day 20 ± SD	Day 30 ± SD
Friction style	Chemical	32.67 ± 0.47	31.05 ± 0.23	30.66 ± 0.63	31.39 ± 0.47
Friction style	Mechanical	33.44 ± 0.23	29.56 ± 0.84	32.33 ± 0.42	32.39 ± 0.41
Friction style	Combined	32.28 ± 0.23	29.72 ± 0.40	29.55 ± 0.42	29.72 ± 0.31
Friction style	Autoclave	32.33 ± 0.13	30.11 ± 0.41	30.33 ± 061	29.94 ± 0.412
Spring style	Chemical	33.05 ± 1.61	30.44 ± 0.81	29.22 ± 0.93	31.61 ± 0.62
Spring style	Mechanical	35.44 ± 1.81	30.88 ± 0.41	30.77 ± 0.42	30.97 ± 0.51
Spring style	Combined	44.22 ± 1.55	29.78 ± 0.71	30.50 ± 0.81	29.55 ± 0. 64
Spring style	Autoclave	41.72 ± 2.41	31.56 ± 0.71	31.06 ± 0.94	30.83 ± 0.73

**Table 3:** Hex and conical connection bacterial penetration values before aging (control values) and after aging. PCR bacterial penetration quantification values of each implant-abutment complex, before and after torque controller aging, N = 24, *P* = 0.0301.

Bacteria	Device	Aging Method	Abutment	No. of Penetrated Bacteria—HEX—Control	SD	No. of Penetrated Bacteria—HEX—Final	SD	Abutment	No. of Penetrated Bacteria—Conical—Control	SD	No. of Penetrated Bacteria–Conical—Final	SD
Pg	Spring style	Chemical	HEX	201	34.32 ± 0.72	1,563,004	26.07 ± 0.04	Conical	23,841	31.9 ± 0.36	86,580,914	20.46 ± 0.04
Pg	Spring style	Chemical	HEX	2694	30.21 ± 0.10	2,609,534	25.35 ± 0.06	Conical	371,311	28.07 ± 0.11	33,972,949	21.77 ± 0.01
Pg	Spring style	Chemical	HEX	N/A	>35 **	1,863,102	25.82 ± 0.16	Conical	909,712	26.82 ± 0.19	9,864,718	23.49 ± 0.05
Pg	Spring style	Mechanical	HEX	2537	30.31 ± 0.02	1,035,009	26.64 ± 0.01	Conical	174,294	29.12 ± 0.10	252,850,757	18.97 ± 0.07
Pg	Spring style	Mechanical	HEX	904	31.94 ± 0.66	21,939,182	22.38 ± 0.03	Conical	169,975	29.16 ± 0.14	24,168,534	22.25 ± 0.05
Pg	Spring style	Mechanical	HEX	N/A	>35 **	5,845,369	24.23 ± 0.02	Conical	936,175	26.78 ± 0.01	6,893,161	24.00 ± 0.01
Pg	Friction style	Combined	HEX	1320	31.34 ± 0.38	4,748,162	24.52 ± 0.04	Conical	678,043	27.23 ± 0.07	5,441,000	24.33 ± 0.09
Pg	Friction style	Combined	HEX	443	33.07 ± 0.71	48,098,276	21.29 ± 0.04	Conical	1,803,958	25.86 ± 0.02	108,515,532	20.15 ± 0.16
Pg	Friction style	Combined	HEX	567	32.68 ± 0.04	214,570	28.84 ± 0.06	Conical	25,871,815	22.15 ± 0.09	52,044,650	21.18 ± 0.04
Pg	Friction style	Autoclave	HEX	921	31.91 ± 0.14	103,279	29.86 ± 0.46	Conical	2,134,960	25.63 ± 0.15	12,769,220	23.14 ± 0.02
Pg	Friction style	Autoclave	HEX	493	32.90 ± 0.47	3,305,998	25.02 ± 0.11	Conical	22,658,459	22.33 ± 0.26	21,860,685	22.39 ± 0.01
Pg	Friction style	Autoclave	HEX	362	33.39 ± 0.98	5,499,823	24.31 ± 0.25	Conical	11,843,347	23.24 ± 0.12	16,004,206	22.82 ± 0.27
Fn	Friction style	Chemical	HEX	10,330	28.08 ± 0.10	2,198,798	19.59 ± 0.01	Conical	N/A	>35 **	8,221,682	17.50 ± 0.11
Fn	Friction style	Mechanical	HEX	4449	29.42 ± 0.18	2,632,031	19.30 ± 0.07	Conical	1322	31.33 ± 0.18	370,979	22.41 ± 0.08
Fn	Friction style	Mechanical	HEX	7164	28.66 ± 0.08	18,113	27.19 ± 0.10	Conical	2226	30.50 ± 0.03	1,356,854	20.35 ± 0.04
Fn	Friction style	Mechanical	HEX	655	32.45 ± 0.01	3,631,275	18.79 ± 0.03	Conical	430	33.11 ± 0.15	15,020,465	16.54 ± 0.01
Fn	Spring style	Combined	HEX	2989	30.05 ± 0.05	360,593	22.45 ± 0.03	Conical	587	32.61 ± 0.02	1,054,171	20.75 ± 0.14
Fn	Spring style	Combined	HEX	2404	30.39 ± 0.06	207,596	23.33 ± 0.18	Conical	N/A	>35 **	900,321	21.00 ± 0.06
Fn	Spring style	Combined	HEX	474	32.97 ± 0.09	1,593,739	20.10 ± 0.02	Conical	3806	29.65 ± 0.007	1,365,443	20.34 ± 0.18
Fn	Spring style	Autoclave	HEX	7752	28.54 ± 0.02	17,774	27.22 ± 0.01	Conical	548	32.72 ± 0.007	7,574,143	17.63 ± 0.06
Fn	Spring style	Autoclave	HEX	1438	31.21 ± 0.22	2,269,280	19.54 ± 0.06	Conical	438	33.08 ± 0.24	5,057,527	18.27 ± 0.05
Fn	Spring style	Autoclave	HEX	248	33.99 ± 0.41	1,774,216	19.93 ± 0.05	Conical	717	32.3 ± 0.01	1,199,754	20.55 ± 0.09
Fn	Friction style	Chemical	HEX	849	32.04 ± 0.21	1,735,460	19.96 ± 0.01	Conical	1623	31.00 ± 0.07	745,042	21.30 ± 0.03
Fn	Friction style	Chemical	HEX	1743	27.06 ± 0.04	19,724	27.06 ± 0.04	Conical	246	33.99 ± 0.47	1,257,901	20.47 ± 0.04

** Values greater than 35 are considered below quantification limit.

**Table 4:** Four-way ANOVA demonstrated bacteria penetration to IAC regardless of connection and type of torque controller.

**Variable**	**DF**	**Sum sq.**	**Mean sq.**	**F-Value**	**Pr (>F)**
Bacteria	1	7.492 × 10^15^	7.492 × 10^15^	5.050	0.0301 *
Connection	1	5.090 × 10^15^	5.090 × 10^15^	3.431	0.0712
Controller	1	1.187 × 10^15^	1.187 × 10^15^	0.800	0.3762
Chemical a.	3	3.301 × 10^15^	1.100 × 10^15^	0.742	0.5333
Residuals	41	6.082 × 10^16^	1.483 × 10^15^		
**Variable**	**DF**	**Sum sq.**	**Mean sq.**	**F-Value**	**Pr (>F)**
Bacteria	1	7.492 × 10^15^	7.492 × 10^15^	5.050	0.0301 *
Connection	1	5.090 × 10^15^	5.090 × 10^15^	3.431	0.0712
Controller	1	1.187 × 10^15^	1.187 × 10^15^	0.800	0.3762
Chemical a.	3	3.301 × 10^15^	1.100 × 10^15^	0.742	0.5333
Residuals	41	6.082 × 10^16^	1.483 × 10^15^		

* *P* < 0.05.

**Table 5:** Linear regression presented more *P. gingivalis* penetration with more effect on conical IAC regardless of the aging process.

	Estimate	Std. Error	*t* Value	Pr (>|*t*|)
Intercept	33,912,159	14,708,550	2.306	0.0263 *
*P. gingivalis*	−24,986,453	11,118,619	−2.247	0.0301 *
Conical c.	20,594,575	11,118,619	1.852	0.0712
Friction t.	−9,946,210	11,118,619	−0.895	0.3762
Chemical a.	−13,986,462	15,724,102	−0.889	0.3789
Autoclave a.	−23,082,246	15,724,102	−1.468	0.1497
Combined a.	−9,842,443	15,724,102	−0.626	0.5348

* *P* < 0.05.

## Data Availability

Data is contained within the article.
